# When timing matters—misdesigned dam filling impacts hydropower sustainability

**DOI:** 10.1038/s41467-021-23323-5

**Published:** 2021-05-24

**Authors:** Marta Zaniolo, Matteo Giuliani, Scott Sinclair, Paolo Burlando, Andrea Castelletti

**Affiliations:** 1 Department of Electronics, Information, and Bioengineering Politecnico di Milano, Milano, Italy; 2 Institute of Environmental Engineering, ETH Zurich, Zurich, Switzerland

**Keywords:** Environmental impact, Hydrology

## Abstract

Decades of sustainable dam planning efforts have focused on containing dam impacts in regime conditions, when the dam is fully filled and operational, overlooking potential disputes raised by the filling phase. Here, we argue that filling timing and operations can catalyze most of the conflicts associated with a dam’s lifetime, which can be mitigated by adaptive solutions that respond to medium-to-long term hydroclimatic fluctuations. Our retrospective analysis of the contested recent filling of Gibe III in the Omo-Turkana basin provides quantitative evidence of the benefits generated by adaptive filling strategies, attaining levels of hydropower production comparable with the historical ones while curtailing the negative impacts to downstream users. Our results can inform a more sustainable filling of the new megadam currently under construction downstream of Gibe III, and are generalizable to the almost 500 planned dams worldwide in regions influenced by climate feedbacks, thus representing a significant scope to reduce the societal and environmental impacts of a large number of new hydropower reservoirs.

## Abstract (web summary)

Filling of dams, when coinciding with droughts, can lead to severe downstream hydrology and ecology problems. The authors hence here provide a multisectoral perspective, using a dam in Ethiopia as an example, to develop adaptive filling solutions that support decision making, favourable filling timing and an effective filling policy.

## Introduction

Hydropower is the dominating renewable electricity source worldwide, accounting for the largest share of energy production and investments allocated on new projects^1^. However, hydropower dam development does not occur without environmental and social costs^2,3^. Efforts towards sustainable dam planning have addressed strategic dam sizing^4^, dam location^5,6^, and basin-wide portfolios^7–9^ to minimize long-term impacts of such infrastructures. Yet, before starting electricity production, dam reservoirs must be filled withholding a substantial fraction of the river streamflow from downstream users. The rate at which a reservoir is filled has direct implications on potential conflicts between upstream and downstream interests. In this phase, precaution towards downstream impacts requires transiting high percentages of inflow, resulting in multiyear, even decadal, filling transients^10^. Conversely, upstream interests (e.g., hydropower production) favor fast impoundment of water, which can generate critical periods of minimal streamflow downstream. Increasingly variable hydroclimatic regimes characterized by strong interannual oscillations present an additional challenge in the design of filling strategies as the same policy can yield very different results depending on whether it occurs during a wet or a dry spell.

Historically, the filling of large dams has generated serious international tensions. In the Middle East, threats of an armed conflict were raised in 1992, when the filling of the Turkish Atatürk Dam on the Euphrates cut the water flow to downstream Syria and Iraq by 75%^11^. In 2019, the filling of the Ilsu dam on the nearby Tigris reinflamed tensions in the Middle East, in the midst of their unprecedented water, and humanitarian, crisis^12^. Similar transboundary tensions were generated by the filling of Gibe III, the “most controversial dam in Africa”^13^, located in the Omo-Turkana Basin (OTB) shared by Ethiopia and Kenya. After the Gibe III dam began impounding water (2015–2016), an upsurge of local and international groups contested the insufficiency of the summer flood pulse necessary to support downstream riparian activities, as well as a 2 m level drop in the downstream Kenyan lake Turkana^14,15^. Perhaps the most controversial case, given its global resonance, is the filling of The Grand Ethiopian Renaissance Dam (GERD) on the Blue Nile. In 2020, at the beginning of the tenth year of negotiations, there is still no international filling agreement between Egypt, downstream, demanding guarantees on minimum GERD releases, and Ethiopia, upstream, resolved to maintain discretion on its operations^16–18^.

State-of-the-art efforts on cooperative filling consider static filling strategies designed to impound (or release) fixed fractions of inflow or absolute water volumes determined on average hydrological conditions, and explore how hydrological variability, climate change^19,20^, coordination between co-riparian countries^21^ impact filling outcomes. The focus of such studies ranges from the analysis of engineering constraints^22^ and stability^23^ to ecosystem services^24^ and water-energy-food nexus^25^. Results show that in general the filling outcomes are largely determined by hydroclimatic variability: if the filling occurs during a drought, enhanced impacts are experienced by all downstream sectors. The novelty of this work is in the introduction of adaptivity in the filling operations by informing them with seasonal and multi-annual drought forecasts, in order to identify both a favorable filling timing, i.e., when to start the filling, and an effective filling policy, i.e., how to fill the reservoir by timely adjusting the filling rate in anticipation of a wet spell or a drought emergency^26^ driven by global climate oscillations. Accordingly, we demonstrate the framework with a retrospective analysis of the recent filling of Gibe III. The reference provided by the contested historical filling of the reservoir allows investigating the potential of these adaptive solutions in addressing the tradeoff between upstream and downstream competing interests, along with quantifying the role of hydroclimatic variability.

We find that the Gibe III filling impacts were disproportionally amplified by an ongoing drought, and show how a more favorable dam filling timing can be inferred in advance, by monitoring long-term climatic oscillations in the basin. Once the optimal timing is established, adaptive filling policies can be designed for better responding to natural hydroclimatic variability, thereby minimizing downstream flow alterations without damaging hydropower production levels. A new megadam, Koysha, with a 9 billion cubic meter reservoir capacity, is currently under construction downstream of Gibe III and is expected to begin filling in 2021 in conjunction with another multi-year dry spell. Our results suggest that the impacts of this new project will be amplified by these unfavorable hydroclimatic conditions, potentially impacting the political and social stability within the region.

## Results

### Behind the filling controversy

In recent years, Ethiopia’s domestic electricity demand has witnessed a dramatic increase, propelled by an unprecedented growth in its GDP^27^. Yet, Ethiopia’s plans for the electricity sector in the near future are even more ambitious. By 2025, the country is striving for 100% electricity access^28^, a 10-fold increase in power generation capacity since 2013 that would not only cover internal demand, but also allow a substantial electricity export^29^ and a fully decarbonized economy^30^.

The key to becoming the green battery of Africa is accessing its exceptional renewable resource potential estimated around 60 GW of electric power from hydropower, wind, solar, and geothermal sources^31^, of which hydropower represents the largest share (45 GW)^32^. The Ethiopian Electric Power Corporation has thus embarked on an ambitious dam building program intended to exploit its abundant water reserves^33^. Among the mega-infrastructures recently built or under construction we count the GERD, on the Blue Nile^10^, along with Gibe III and Koysha, on the Omo river. Gibe III, commissioned in 2015, doubled Ethiopian hydroelectric installed hydropower capacity and has the potential to significantly alter Omo’s streamflow regime with its massive reservoir volume of 14.7 billion m^3^, corresponding to the average yearly river flow at dam site. Differently to the other mega-infrastructures, Gibe III is already completed and currently operating at regime conditions, thus allowing to benchmark alternative filling strategies against historical operations.

**Fig. 1:**
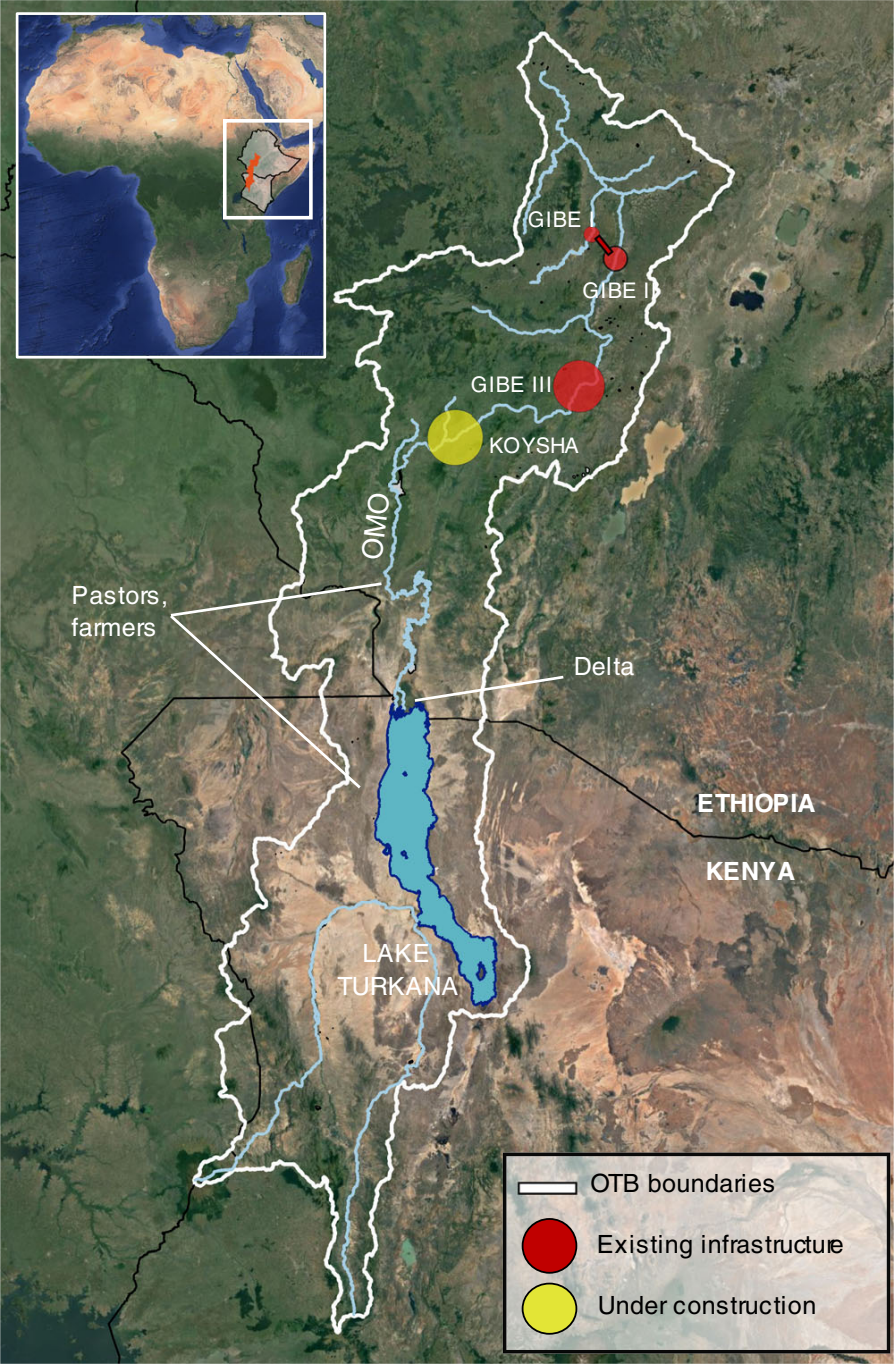
Geography of the Omo-Turkana Basin (OTB). The Omo river collects the abundant rainfalls of the Ethiopian highlands and flows southwards through the Omo valley contributing about 90% of the annual inflow to Lake Turkana, where its outlet forms a complex delta across the Ethiopian-Kenyan border. About 500 thousand pastoralists and farmers inhabit the area depending on the Omo or Turkana waters for their livelihood^34^. The Gibe-Koysha dam cascade regulates the river hydrology, comprising Gibe I and II, the recently completed Gibe III, and the Koysha dam currently under construction. Marker size is proportional to the installed hydropower capacity. Basemap: Google Satellite, Map data ©2015 Google.

The Omo river is one of the largest and steepest Ethiopian rivers, and was a main target of dam expansion given its remarkable reserve of unharnessed hydropower potential. It originates in the Ethiopian Shewan highlands, and streams southwards through a mountainous area before slowing its pace as it meanders in the lower Omo valley (Fig. 1). At the Ethiopian-Kenyan border, the river forms an extensive delta and contributes about 90% of the inflow to Lake Turkana, an endorheic lake of the Kenyan Rift Valley, and the world’s largest desert lake^34^.

A three-season meteorological year characterizes the regional climate, a rainy Kiremt season (June-September) contributing the bulk of the annual precipitation through intense convective storm events, a dry Bega season (October-January) carried by Arabian desert winds, and a milder wet Belg season (February–May) induced by a wet air mass coming from the Indian Ocean^26^. In addition to seasonal variability, a marked interannual climate variability affects the region, as a result of the influence of large-scale oscillation patterns in the atmospheric-ocean system^35,36^. Such teleconnections are responsible for frequent severe drought episodes recurring every 5 to 10 years that cause widespread water shortages in the country, with negative societal effects, for example the catastrophic Ethiopian famine of the mid 1980s^37,38^.

The Omo river hydrology is characterized by a late summer flow peak that conveys the Kiremt rainfall, and reaches about 1000 m^3^/s in the lower valley. Local ecosystems and activities largely depend on this flood pulse that enables recession agriculture practices and replenishes grazing lands for livestock, supporting the livelihood of about 200,000 people in Southern Ethiopia^34,39^. Reaching Lake Turkana, the flood pulse sustains a biodiverse delta, and produces lake level oscillations that are vital to nutrient circulation, fish spawning, and the regeneration of lake shore grazing area for livestock, a crucial protein source for the 300,000 people inhabiting the poorest region in Kenya^40^.

A series of dams and hydropower schemes was built on the river, including Gibe I (187 MW), Gibe II (420 MW), and Gibe III (1870 MW). The dam cascade will be concluded with the addition of Koysha (2160 MW) currently under construction and expected to be completed in 2021. Since the Gibe III project was made public in 2009, it received opposition regarding the inadequacy of its Environmental Impact Assessment in capturing the dam’s downstream alterations^40,41^, and the depth of its potential social and political impacts^39^, and an unprecedented upsurge of national and international criticism erupted since the reservoir behind the dam started to impound water^13^. Reports say that in 2015 and 2016, the flood pulse downstream the dam did not occur or was severely dampened, and thus inadequate to serve its functions^15,42^, dramatically damaging the river-related ecosystems and activities downstream the dam relying on it^43,44^. Simultaneously, during Gibe III filling, Lake Turkana level dropped 1.7 m, of which over 1 m in the first year^34^. Were these dramatic impacts the inevitable price to pay for dam development, or was a (more) sustainable filling possible?

**Fig. 2:**
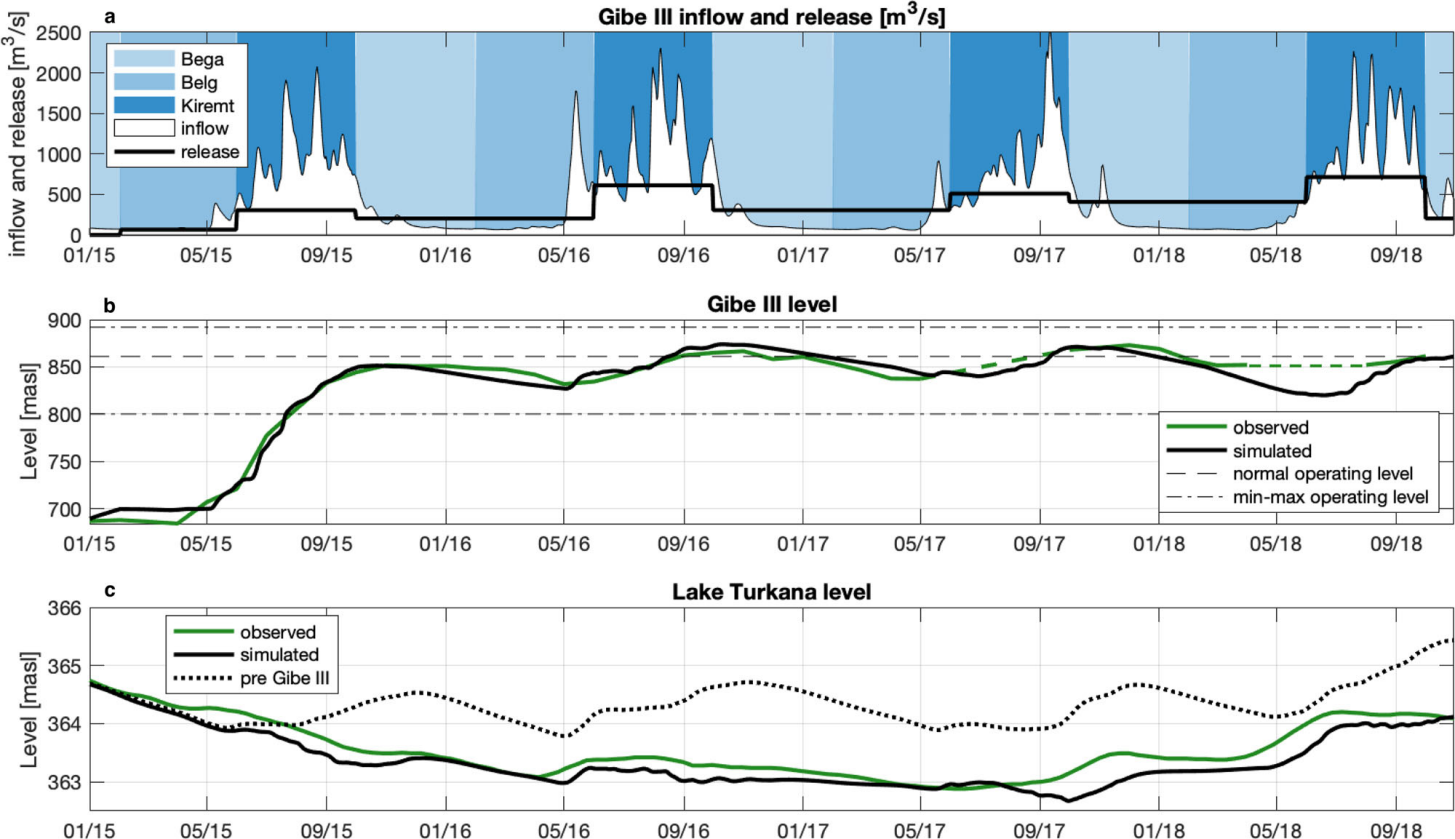
Reconstructed historical filling strategy. **a** Gibe III reservoir reached its normal operating level within its first two years of operations by impounding the near totality of the 2015 Kiremt season inflow, and a significant fraction of 2016’s. **b** In the two following years, Gibe III level oscillates around its operational level as a consequence of a release pattern that increases low flows and reduces high flows with respect to natural Omo hydrology. **c** Simultaneously, Lake Turkana suffered a 2-m level drop with respect to a simulation of a scenario in which Gibe III was not built. While the Lake Turkana level trajectory estimated from satellite altimetry is publicly available^64^, we reconstructed the Gibe III level trajectory from Sentinel 2 image classification (see “Methods”).

To address this question, we analyzed the historical filling strategy and explored alternative options by changing both filling timing and operations. Since no official record of Gibe III operations during the filling is publicly available, we first reconstructed the historical strategy using satellite imagery and a simulation model of the OTB (see “Methods” and Supplementary Fig. 2). Overall, the reconstructed system dynamics (Fig. 2) is coherent to news reports^15,42^, showing the largely impounded 2015 and 2016 Kiremt season streamflow, a fast level increase in Gibe III, and a steep drop in Lake Turkana level during the initial dam filling.

### The role of timing in determining filling impacts

**Fig. 3:**
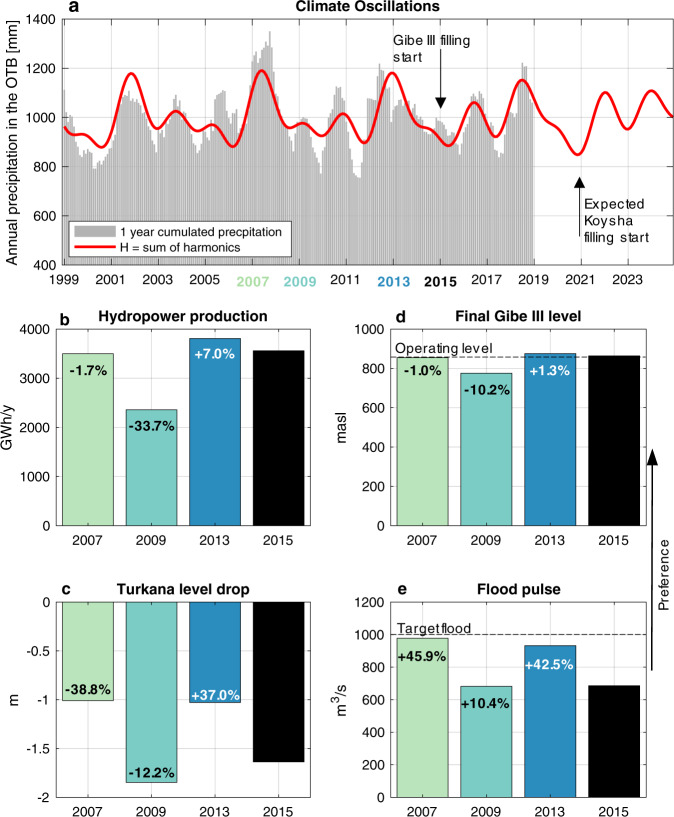
Climatic oscillations can inform a favorable timing for filling. A pattern of harmonic climatic oscillations governs the magnitude of annually cumulated rainfall occurring on the OTB, shown at a monthly time step (**a**). Panels (**b**–**e**) contrast the historical 2015 filling performance (black bar) with alternative filling timings (colored bars) with respect to relevant indicators. The bar labels report the percentage change in the value of the indicator with respect to the historical performance. Filling Gibe III reservoir during an upwards phase of water availability (e.g., 2013), instead of a downwards phase as historically, could have resulted in a more efficient, and less conflictual filling. By projecting the harmonic trends into the future, we advise to delay Koysha filling by one year and begin in 2022 instead of the planned 2021, as the additional stress caused by a bad timed filling stress could have detrimental consequences on the fragile social and ecological balances of the region.

To understand the role of timing (i.e., when dam filling is initiated), we performed a retrospective analysis by simulating the reconstructed historical filling policy and assuming this took place in different years featuring diverse hydroclimatic conditions. The annual cumulated precipitation in the basin from 1999 to 2018 shows a clear multiyear climatic oscillation that can be well approximated by the sum of three harmonics (Fig. 3), associated with the ocean-atmospheric interactions insisting in the region (see “Methods”). Gibe III filling began in 2015, at the negative peak of a prolonged downwards phase in precipitation abundance; intuitively, this represented an unfortunate timing to rapidly impound a large water volume into a reservoir.

Looking at the climatic oscillations, we analyzed alternative timings corresponding to upwards (2007 and 2013) and downwards (2009 and 2015, which is the historical starting date) phases in precipitation abundance. Results show that the filling outcomes are strongly determined by the harmonic phase in which the filling starts (Fig. 3b–e). According to all considered indicators (see “Methods” and Supplementary Information for details about their formulation) reflective of both upstream interests (i.e., hydropower production, final Gibe III level) and downstream preservation (i.e., drop in Lake Turkana level, flood pulse magnitude), the worst timing to initiate dam filling would have been 2009, which corresponds to the onset of a multi-year dry spell. Conversely, starting the filling in 2013, would have benefited all sectors involved and contained the sharp intersectoral conflict observed in 2015. In particular, 2013 would have favored upstream water users yielding additional 124 GWh/year in hydroelectricity, corresponding to the electricity demand of 620 thousand Ethiopians at 2017 consumption rate^45^, or to an annual revenue of 8.68 Million USD assuming the electricity was sold to Kenya at the agreed price of 0.07 USD/kWh^46^. In addition, the lake Turkana level drop could have been reduced to 1 m instead of 1.63 m, and the flood pulse magnitude increased by 42% with respect to that actually observed. An extended analysis including additional alternative filling timings is proposed in Supplementary Fig. S6.

This analysis shows that, in the future, the projection of the harmonic trends of precipitation could usefully inform a forward-looking planning of the timing of Koysha construction, in order to synchronize filling to a wet spell, rather than aggravating the expected natural water scarcity situation following a dry spell. Koysha filling is expected to start in 2021, again at the bottom of a steep decline in precipitation foreseen in the 2 previous years, thus likely magnifying the stress of a long-running water shortage. Instead, beginning the filling one year later, at the inversion of the precipitation trend, would significantly reduce the impact downstream and produce benefits upstream.

### Forecast-informed adaptive filling

Our historically reconstructed reservoir operations during and after the Gibe III filling phase, do not achieve the target annual flood pulse even when the timing to start filling is favorable, and all cases result in a significant drop in Lake Turkana level (Fig. 3c and e). These shortcomings motivate searching for alternative, adaptive filling strategies for better responding to the seasonal hydroclimatic variability.

Taking advantage of advanced Machine Learning and data mining techniques, we synthesize global datasets of climate oscillations (Supplementary Table 1) into a compact drought index forecast, namely the Standardized Precipitation and Evaporation Index (SPEI), which is representative of upcoming hydro-meteorological anomalies at the Omo-Turkana basin scale (see “Methods” and Supplementary Fig. 3). Adaptive filling policies use the forecasted drought index to speed up the filling process during wet spells, and, conversely, increase releases during dry seasons would sustain downstream activities (see “Methods”).

**Fig. 4:**
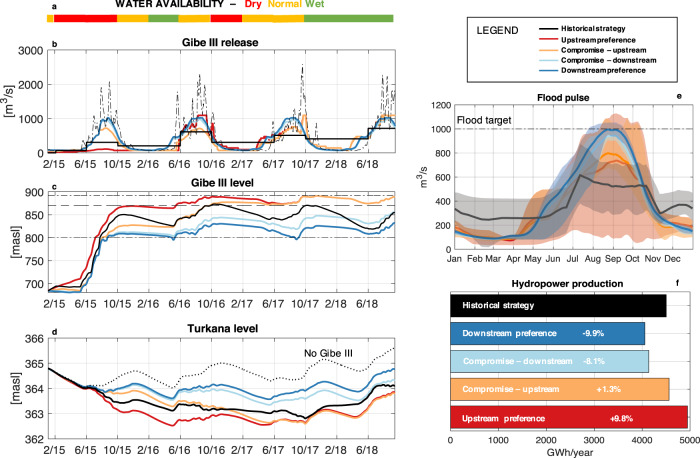
Adaptive filling strategies can reduce filling impacts. The seasonal forecasts of Standardized Precipitation and Evaporation Index expressed in terms of dry, normal, and wet conditions with respect to seasonal average (**a**) inform the designed adaptive filling strategies (**b**, **c**). Different colors correspond to adaptive strategies with different tradeoffs between upstream and downstream competing interests, blues for more environmental inclined, and reds for hydropower inclined strategies, while the historical strategy is represented in black. Adaptive strategies demonstrate the ability to significantly reduce downstream impacts on Lake Turkana (**d**) and average river hydrology (**e**, where the shaded areas refer to the interannual variability) while remaining within a contained range of historically produced hydropower. The numerical labels in the barplot of **f** quantifies the percentage difference in hydropower production associated with adaptive strategies normalized to the historical production. This figure illustrates 4 representative adaptive solutions, compared to the historical solution in black. The complete set of adaptive policies resulted from the optimization is reported in Supplementary Fig. 4.

A total of over one hundred adaptive filling strategies were designed to provide a thorough exploration of the basin sectoral tradeoffs (see the “Adaptive Filling Strategies” section of the “Methods” for details on their design). Four representative informed strategies are visualized in Fig. 4 and compared with historical operations, assuming to begin the filling in 2015 in all cases, for comparability. The Standardized Precipitation and Evaporation Index (SPEI) seasonal forecasts (Fig. 4a) confirm that Gibe III filling started during a drought, but water availability conditions improved towards mid-2017. Tradeoffs are evident between upstream and downstream interests, whereby strategies attaining high hydropower production are also associated with large negative impacts downstream. Notably, the Downstream Preference policy ensures high Gibe III releases (panel b) especially in the first years (2015–2016), nearly halving lake Turkana level drawdown with respect to observed conditions (panel d) and preserving the natural flood pulse in the delta (panel e). The average river streamflow of the wettest 10 consecutive days ((i.e., the approximate length of the flood peak in pristine river conditions^41^) in the year under this policy reaches 1130 m^3^/s. However, this policy is estimated to produce a 9% lower hydropower production with respect to the historical one, corresponding to the electricity demand of 2.2 Million Ethiopians^45^, or, if the electricity was sold to Kenya, a lost revenue of 28.3 Million USD per year in the first four years. Conversely, the Upstream Preference policy surpasses the historical hydropower production (+30.9 Million USD/year, or the demand of 2.21 Million Ethiopians) by implementing a fast filling that reaches Gibe III operating level within the first year, at the cost of significant alterations on Lake Turkana levels. Interestingly, the Omo streamflow in the wettest 10 days of the year averages 900 m^3^/s, significantly lower than the Downstream preference, yet, 28% higher than historically observed. Finally, the Compromise-upstream policy achieves a historically equivalent hydropower production, while maintaining a significantly more natural hydrology downstream in terms of flood pulse, which is, on average, nearly 300 m^3^/s higher than historically observed during the expected peak in late August.

The entire ensemble of adaptive policies produced (Supplementary Fig. 4) thoroughly explores the space of compromises and tradeoffs between the conflicting interests coexisting in the OTB. Hydropower production of adaptive policies ranges from −12% to +9.7% with respect to historically observed. The drop in lake Turkana measured between January 2015 and November 2018 levels ranges from 1.2 m (comparable to the historical 1.1 m) to just a few centimeters drop yielded by policies favoring a slower reservoir filling. Interestingly, the average magnitude of the flood pulse in late August, historically just over 500 m^3^/s, is considerably improved by the entire ensemble of adaptive policies, that obtain a minimum of 720 m^3^/s up to 1000 m^3^/s. Overall, by considering the entire range of adaptive strategies we notice the potential to largely contain environmental alterations with a comparatively small loss in hydropower production. Moreover, downstream alterations can be partially contained even without compromising any electricity production.

Notably, the numerical results obtained here refer to a scenario in which the filling timing is not adjusted. We can expect a further reduction in upstream-downstream conflicts if the implementation of an adaptive filling policy is paired with a better timed filling.

## Discussion

**Fig. 5:**
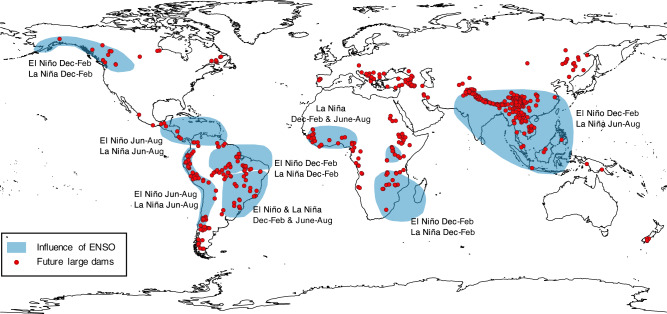
Future dams overlap regions with a strong ENSO influence. The red points indicate the locations of medium-to-large future hydropower reservoirs and dams, extracted from the FHReD database^1^. Dam height is generally employed to discern between small, medium, and large dams, but in the absence of this information, we consider as medium-to-large the hydropower projects with an installed capacity grater than 150 MW, retaining a total of 642 dams of the over 3700 reported in the database. A blue shade highlights the regions of the globe that are most affected by El Niño and La Niña oscillations^76^. Over 70% of medium-to-large future dams are located in areas affected by the ENSO teleconnection.

Sustainable dam planning has paved the way towards a more socially and environmentally inclusive hydropower development that focused on limiting dam-induced socio-environmental costs during dams regime operations^47–50^. Yet, the initial filling phase of a dam can generate critical impacts by withholding in the reservoir a substantial fraction of the river streamflow, and significantly reducing downstream water availability. Hydrological variability can play a key role in magnifying or containing the stress of filling: if the filling occurs during a dry spell, the basin is further exposed to water shortage and intersectoral tensions. This is the case analyzed in this paper, where a rapid filling of the reservoir irresponsive to variations in the water availability paired with an ongoing drought inducing Gibe III association with the label of “most controversial dam in Africa”^13^. In the proposed retrospective analysis, we demonstrate that the investigation of climate oscillations could have informed a more favorable filling timing, as well as adaptive filling operations, and significantly contained the associated social and environmental costs. Koysha dam, located downstream Gibe III, is again at risk of synchronizing its filling to an upcoming drought, further endangering the already precarious socio-environmental conditions of the Omo-Turkana Basin. Despite these quantitative results refer to this specific case study, they may entail some patterns that have a high chance to be common across systems to which the novel approach and tools we propose in this work can be generalized. Currently, of the nearly 650 medium to large dams under construction in the world^1^, 70% are being built in regions under the influence of the El Niño Southern Oscillation, the prevalent global interannual signal of climate variability^51,52^ (Fig. 5). In these areas, teleconnection analysis has the potential to increase our predictive skills in anticipating hydrological variability in the medium-to-long term, which can be exploited to minimize filling impacts. Given the unprecedented global dam expansion envisioned for the coming years, we consider the use of tools and methods such as those presented here to be both generally applicable, and beneficial for enhancing the sustainability of hydroelectric dams operations during the critical initial filling phase.

It is, however, important to consider that while on the one hand filling a dam during a dry year can jeopardize water-related activities and overall basin stability, on the other hand, postponing dam filling will generate repercussions on the immediate project’s energy generation capacity and expected economic return. Additionally, inferring a favorable filling timing by projecting past climatic trends in the future is associated with a level of uncertainty enhanced by ongoing climate change trends. It is thus recommended to consider a portfolio of alternative renewable energy sources (e.g., solar, wind, geothermal, biomass, and tides), in addition to hydroelectricity, that can compensate the delay in hydropower production possibly entailed by a sustainable filling strategy.

## Methods

### Omo-Turkana Basin model

The model of the Omo-Turkana Basin relies on a combination of TOPKAPI-ETH^53,54^, a spatially distributed hydrological model, with a dynamic, conceptual model of Gibe III, the lower Omo Valley, and lake Turkana.

TOPKAPI-ETH is a spatially distributed hydrological model that extends the original TOPKAPI rainfall-runoff model^55^, particularly in respect of simulating anthropogenic influences on the catchment water balance. The model performs a spatial and temporal representation of the main hydrological processes at the basin scale, accounting for runoff generation, routing, evapotranspiration, also including snow and glacier dynamics when necessary^56^. Spatial heterogeneity of the OTB is represented by discretizing the domain with a regular grid of 1 km^2^ of resolution, while the temporal dynamics is characterized at a daily time step. The model inputs are daily values of precipitation, temperature, and cloud cover; the model outputs are Gibe III inflows, lateral contributions in the Lower Omo valley (between Gibe III and lake Turkana), and the additional inflows to lake Turkana provided by the Turkwel and Kerio rivers in Kenya. Daily rainfall estimates are available from the TAMSAT archive with a 4 km resolution for the African continent^57^ at https://www.tamsat.org.uk/, and satellite-based temperature and cloud cover records from MERRA-2 at a resolution of 0.5^∘^ × 0.625^∘^^58^ at https://gmao.gsfc.nasa.gov/reanalysis/MERRA-2/.

With regards to the strategic model employed to simulate filling strategies, the daily dynamics of Gibe III and lake Turkana is described by the mass balance of their water volumes, where the release volume of Gibe III is determined by the simulated filling policies, followed by a regime policy activated when the filling has completed (i.e., when the level of Gibe III reaches the normal operating level equal to 851 masl). Geomorphological and technical characteristics of Gibe III reservoir, dam, and power plant are published in the project’s impact assessment^59^ available for download at https://afdb.org/sites/default/files/documents/environmental-and-social-assessments/g3_esia.pdf. Lake Turkana, instead, is an endorheic lake, and the only water output is due to evaporation. According to the daily time-step adopted in the model, the reach of the Omo river downstream from Gibe III is modeled as a plug-flow canal in which the velocity and direction of flow are constant. A transit lag time of 16 days from Gibe III to lake Turkana is estimated from the TOPKAPI-ETH simulations.

### Adaptive filling strategies

Our proposed adaptive filling policy determines the dam release in a given day as a function of the cyclostatonary average streamflow for that day prior the dam construction. Specifically, the natural streamflow is scaled in proportion to the expected hydrological conditions for the incoming season according to the forecast of the Standardized Precipitation and Evaporation Index (SPEI) drought index. In this formulation, the three scaling factors associated with the three classes of SPEI (i.e., dry, normal, wet) are the decision variables of the filling optimization problem; we searched the optimal value of these factors with respect to the problem’s objectives by using the self-adaptive Borg Multi-Objective Evolutionary Algorithm^60^. The Borg MOEA has been shown to be highly robust across a diverse suite of challenging multiobjective problems where it met or exceeded the performance of other state-of-the-art MOEAs^61–63^. The multiobjective nature of this algorithm enables the generation of the entire set of alternative filling policies that explore the tradeoffs between the three objective functions of the filling problem: hydropower production, environmental deviations, and Gibe III level (See Supplementary Fig. 4). Once the filling is completed with a given filling policy, the reference dam regime operation is implemented. Such operation is designed via Stochastic Dynamic Programming (Bellman, 1957) with the objective of maximizing hydropower production.

The three objective functions, representing the main upstream and downstream interests, were formulated through a participatory process involving key stakeholders active in the system, that participated in dedicated meetings called Negotiation Simulation Labs (NSL) held during the DAFNE research project (http://dafne-project.eu/). During reiterated NSL sessions, ad-hoc objective functions were designed and refined with the help of stakeholders and experts, eventually converging on the maximization of hydropower production at Gibe III, representative of upstream interests, and minimization of the average daily squared distance between the simulated flow in the Omo delta and the annual hydrograph in natural conditions. The latter objective aims at preserving natural hydrology downstream the dam (see Supplementary Fig. 1) which has enabled existing ecosystems and local water-dependent activities (e.g., recession agriculture) to survive and prosper in the last centuries. Moreover, the maximization of the Gibe III level at the end of the filling transient is included in the design of the filling strategy to provide solutions that, for a given hydropower and environmental performance, favor a fuller, rather than unnecessarily emptier reservoir (see the Supplementary Information for the detailed mathematical formulation of these objectives).

The presence of such clearly conflicting interests does not allow the design of a unique optimal solution, but rather a set of non-dominated (or Pareto optimal) solutions. A policy is defined as Pareto optimal if no other solution gives a better value for one objective without degrading the performance in at least one other objective. This 3-objective problem yields an ensemble of over one hundred Pareto-optimal filling strategies, reported in Supplementary Fig. 4. The four strategies extracted from this ensemble and analyzed in Fig. 4 of the main paper are intended to be representative of the difference in optimal filling operations that result from opposite objective preference.

### Historical filling strategy

The historical Gibe III filling strategy (i.e., sequence of dam inflows and releases during the first years) is not publicly available, and was thus reconstructed for the purpose of this study. We derived the sequence of dam releases by assuming that turbines were operated at full capacity (corresponding to maximum efficiency), and the release from the dam was maintained constant within a season. The inflows were obtained from TOPKAPI-ETH hydrological simulations. The simulation of this filling strategy allows the reconstruction of Gibe III and Lake Turkana levels (see Fig. 2). While records of Lake Turkana levels are available in the Database for Hydrological Time Series of Inland Waters (DAHITI, https://dahiti.dgfi.tum.de/en/)^64^, observed Gibe III level data was derived from Sentinel 2 satellite images classification. Sentinel 2 images are available for the area every 5 days at a 30 m spatial resolution^65^ at https://sentinel.esa.int/web/sentinel/sentinel-data-access. The images recorded in the same month are aggregated in the attempt of filtering cloud occlusion. We then performed a land cover classification of the composite images using a combination of NDWI^66^ (Normalized Difference Water Index) and NDVI^67^ (Normalized Difference Vegetation Index) indexes, and derived an estimate of the reservoir surface area from water pixels count (see Supplementary Fig. 2). Using the reservoir bathymetry we finally estimated the corresponding trajectory of the Gibe III level. The coefficient of determination of the simulated filling strategy with respect to the historical observations displayed in Fig. 2 is equal to ${R}^2_{{\rm{GibeIII}}}=0.98$, ${R}^2_{{\rm{Turkana}}}=0.91$.

### Empirical derivation of climatic oscillations

The influence of climate oscillations on Ethiopian meteorology can be decomposed into three contributing phenomena associated with the three oceans^35^. The climatic oscillations shown in Fig. 3 are therefore empirically derived by summing three single term Fourier series *h*^*i*^, *i* = 1, 2, 3 of the form

1 $${h}^{i}(x)={a}_{0}+{a}_{1}* \cos (x* w)+{b}_{1}* \sin (x* w)$$

where *a*_0_, *a*_1_, *b*_1_, and *w* are the parameters to be calibrated, and *x* is the signal to be approximated. In particular, the first harmonic is specified as *h*^1^(*p*), where the signal *p* is the monthly time series of the annual cumulated precipitation in the OTB. For the second harmonic ${h}^{2}(p^{\prime} )$ the signal to be approximated is computed as the residual precipitation $p^{\prime} =p-{h}^{1}(p)$ that is not captured in *h*^1^(*p*), and analogously, *h*^3^(*p″*) is calibrated on the second residual $p^{\prime\prime} =p^{\prime} -{h}^{2}(p^{\prime} )$. The resulting Pearson correlation coefficient is *ρ* = 0.72.

### Performance of alternative timing of reservoir filling

We investigated the role of filling timing, assessing how the system would have responded to the filling stress if it started in different years. To do so, we simulated the first 24 months of the filling subject to the hydrology of different years. System performance is then evaluated in terms of 4 indicators (see the Supplementary Information for the detailed mathematical formulation):

1. Mean annual hydropower production during the 24 months filling period;
2. Final Gibe III level at the end of the 24 months;
3. Final Turkana level drop referred to the initial lake level;
4. Flood Pulse defined as the average annual maximum flow reaching the delta during the flood season of August-September.

### Seasonal forecasts

To develop season-ahead hydrological forecasts of water availability we use the Climate State Intelligence (CSI) framework^68^, an extension of the Niño Index Phase Analysis^69^, which employs Artificial Intelligence tools to search relevant circulation patterns at the global scale that serve as predictors for meteorological anomalies at the local scale. The CSI framework is articulated in four steps:

1. *Phase distinction:* given a teleconnection signal, the associated teleconnection index is used to group the years in the time horizon into a specified number of phases, that are then evaluated individually. For instance, considering the El Niño Southern Oscillation (ENSO), the MEI index is used to distinguish El Niño and La Niña years, allowing one to uncover possible asymmetries in the effect of a signal on the local scale, e.g., if in a given region El Niño years are associated with a wet spell, La Niña years are not necessarily associated with a dry spell.
2. *Univariate linear forecast:* For each phase of the climate signal, the procedure identifies relevant correlations between a gridded dataset of preseason Sea Surface Temperature (SSTs) and the local variable, retaining SST regions correlated at 95% significance or above. Selected SST regions are then spatially aggregated via Principal Component Analysis (PCA^70^). As in previous applications^68,69^, only the first, most informative, PC is retained as a predictor for a linear forecast model of the local variable *y*:
  2 $${\widehat{y}}_{t}=\beta P{C}_{t-1}+\alpha$$
  A leave-one-out cross-validation is performed to calibrate model coefficients *α* and *β*.
3. *Test of Correlation Significance:* A Montecarlo analysis is run to test the statistical significance of the obtained correlations by randomly shuffling the time series of the local variable to be predicted and repeating the above described steps with unshuffled SSTs and teleconnection index time series.
4. *Multivariate non-linear forecast:* The most informative climate signals for the region of interest are then chosen based on their linear model accuracy and significance. A multivariate non-linear model (Extreme Learning Machine^71^) is then cross-validated on the selected climate signals to produce a data-driven seasonal forecasts of the local variable.

In this analysis, we considered 16 teleconnection signals referred to different time and spatial scales over the 21-year time horizon 1998–2018 dictated by the precipitation data availability. We obtained Global Sea Surface Temperature anomalies from the NOAA’s Extended Reconstructed SST (ERSST) Version 3b, a global monthly gridded dataset with a spatial resolution of 2.5 degrees available at https://www.noaa.gov. From the same source we retrieved the time series of teleconnection indexes. The local variable forecasted is the Standardized Precipitation and Evaporation Index (SPEI) drought index^72^, which has proven to be more effective than the Standard Precipitation Index (SPI) to characterize hot and arid climates, where the evapotranspiration has a key role in depleting the soil moisture and becomes one of the main drivers of a drought^73^. SPEI substitutes the precipitation used for SPI computation with a net precipitation, by substracting the Potential Evapo-Transpiration (PET) estimated from temperature and latitude via Thornthwaite’s method^74^. In this work, the SPEI index within a 6 month cumulation window is used to characterize seasonal water availability in three classes according to a classification commonly used in the literature^75^: dry (SPEI < −0.5), normal (−0.5 < SPEI < 0.5), and wet (SPEI > 0.5). The 6 months time span was selected as frequently used to characterize medium-term hydrological conditions.

Phase specific accuracy of the univariate linear forecast models in cross-validation is reported in Supplementary Table 1, along with the corresponding statistical significance. Balancing accuracy and significance we selected three teleconnection signals, namely the NAO, related to a climatic oscillation that originates in the Atlantic ocean, the PNA, originated in the Pacific Ocean, and SEIO, originated in the Indian Ocean. This choice aligns with the findings in Lanckriet et al.^35^ that demonstrates that three overlapping climatic oscillations each originated in a different ocean contribute to determine the Ethiopian climate. The first Principal Components related to these signals are the inputs of the multivariate Extreme Learning Machine forecast model, which was used in this study to generate a 10 member forecast ensemble. The ensemble average is retained for classifying the upcoming season (see Supplementary Fig. 3).

## Supplementary information

Supplementary Information

## Data Availability

All raw data used in this manuscript are freely available at the following websites. Lake Turkana levels: https://dahiti.dgfi.tum.de/en/. Gibe III dam specifics: https://afdb.org/sites/default/files/documents/environmental-and-social-assessments/g3_esia.pdf. Temperature data: https://gmao.gsfc.nasa.gov/reanalysis/MERRA-2/. Rainfall data: https://www.tamsat.org.uk/. Satellite images for Gibe III surface expansion reconstruction: https://sentinel.esa.int/web/sentinel/sentinel-data-access. Database of future Hydropower Reservoirs and Dams: http://globaldamwatch.org/fhred/. Global Sea Surface Temperature anomalies and teleconnection indexes: https://www.noaa.gov.

## Code availability

Relevant code produced in this work is available in the open source repository 10.5281/zenodo.4587267^77^. The CSI framework is freely available at 10.5281/zenodo.4587283^78^.
