# Supplementary material for: When timing matters—misdesigned dam filling impacts hydropower sustainability
Source: Nat Commun. 2021 May 24;12:3056. doi: 10.1038/s41467-021-23323-5 (PMC8144588; doi:10.1038/s41467-021-23323-5)
Supplement: Supplementary file 1 — Supplementary Information [file 41467_2021_23323_MOESM1_ESM.pdf]

Supplementary Information for: When timing matters - misdesigned dam filling impacts hydropower sustainability

Marta Zaniolo[1], Matteo Giuliani[1], Scott Sinclair[2], Paolo Burlando[2] & Andrea Castelletti[1]

[1]*Department of Electronics, Information, and Bioengineering Politecnico di Milano, Italy.*

[2]*Institute of Environmental Engineering, ETH Zurich, Zurich, Switzerland.*

1 Supplementary Material

System Model The conceptual model of the Omo-Turkana Basin comprises Gibe III reservoir (*GIII*), lake Turkana (*T*), and the Omo river stretch connecting them. A topological scheme representing the system is shown in Supplementary Figure 1.

The dynamics of reservoir and lake storage s_t is modeled as:

$$s_{t+1}^{GIII} = s_t^{GIII} + q_{t+1}^{GIII} - e_t^{GIII} A_t^{GIII} - r_{t+1}^{GIII} \tag{1a}$$

$$s_{t+1}^{T} = s_t^{T} + r_{t+1-lag}^{GIII} + q_{t+1}^{lateral} + q_{t+1}^{Turkwel} + q_{t+1}^{Kerio} - e_t^{GIII} A_t^{GIII} \tag{1b}$$

where s_t^i is the storage to the *i*-th water body (i = GIII, T), e_t^i is the cyclostationary daily evaporation rate, and $A_t^i = a(s_t^i)$ is the reservoir surface estimated from s_t^i given the biunivocal surface-level-storage relation. In the adopted notation, the time subscript of a variable indicates the instant when its value is deterministically known. The release from Gibe III is defined as $r_{t+1}^{GIII} = f(s_t^{GIII}, u_t^{GIII}, q_{t+1}^{GIII})$ where f describes the nonlinear relation between the release decision u_t^{GIII}

and the actual release[1]. The actual release at the end of the time interval is generally equal to the release decision unless physical constraints prohibit it (i.e., if the prescribed release lies outside the minimum and maximum allowable releases, for instance as a result of insufficient water volume in the reservoir, or exceedence of reservoir storage capacity). Lake Turkana, instead, is an endorheic lake, and the only water output is due to evaporation. The lake total inflow is given by the sum of GIbe III release (r_{t+1}^{GIII}), flow of Turkwel ($q_{t+1}^{Turkwel}$) and Kerio rivers (q_{t+1}^{Kerio}), and the lateral contributions in the lower Omo valley ($q_{t+1}^{lateral}$). According to the daily time-step adopted in the model, the river reaches are modelled as plug-flow canals in which the velocity and direction of flow are constant. A transit lag time of $lag = 18$ days is estimated from streamflow data, and corresponds to the average time employed by the water to transit between these two points.

Filling policies are optimized with respect to the following objective functions, defined over the filling horizon HF from January 2015 to October 2018:

- Hydropower Production J^{Hyd} (to be maximized):

$$J^{Hyd} = \frac{1}{Nyears}\left[\sum_{t=1}^{HF} g_t^{Hyd}\right] \tag{2a}$$

where

$$g_t^{Hyd} = \eta\sigma\gamma\bar{h}_t^{GIII} q_t^{turb}; \tag{2b}$$

with $Nyears$ being the number of years in the simulation horizon, η the turbine efficiency, σ the gravitational acceleration, γ the water density, $\bar{h}_t^{GIII}$ the net hydraulic head, and q_t^{turb} the turbinated flow.

- Environmental deviation J^{Env} (to be minimized):

$$J^{Env} = \frac{1}{HF} \sum_{t=0}^{HF-1} g_t^{Env} \tag{3a}$$

where

$$g_t^{Env} = \left(q_t^{natural} - q_t^{delta} \right)^2 \tag{3b}$$

with $q_t^{natural}$ representing the cyclostationary trajectory of natural inflow regime in the Omo Delta estimated from streamflow data prior Gibe III construction (Figure 1), and q_t^{delta} the streamflow reaching the delta for the policy under evaluation. The objective is formulated as the average daily squared distance between these two trajectories over the simulation horizon, and is aimed at preserving natural flow conditions in correspondence to the Omo delta, and consequently a natural inflow pattern in lake Turkana. According to the adopted squared formulation, smaller, and more frequent deviations are preferred to large concentrated deviations with respect to the target.

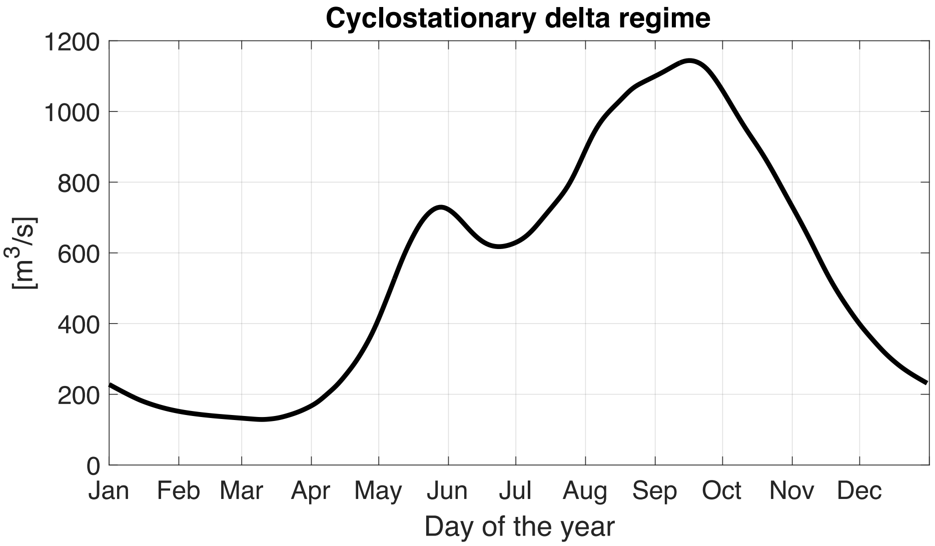

Supplementary Figure 1 Cyclostationary trajectory of natural inflow regime in the Omo Delta estimated from streamflow data prior Gibe III construction.

- Final Gibe III level J^{Fill} (to be maximized and considered in the optimization of filling policies only):

$$J^{Fill} = h_H F^{GIII} \tag{4}$$

where h_H^{GIII} is the reservoir's level at the end of the filling horizon HF.

In the investigation of the role of filling timing, we simulated the first two years of the reconstructed filling strategy assuming to start the reservoir filling in different years. Historically, 2 years were necessary to achieve the normal operating level and, subsequently, the reservoir level remained reasonably constant apart from contained seasonal fluctuations.

To assess the resulting system performance, we used 4 evaluation indicators assessed in a 2-years filling horizon H_{2y} formulated as follows.

1. Annual average hydropower production (i^{Hyd}):

$$i^{Hyd} = \frac{1}{Nyears}\left[\sum_{t=1}^{\mathbb{H}_{\neq\curvearrowright}} \eta g \gamma \bar{h}_t^{GIII} q_{t+1}^{turb}\right] \tag{5}$$

where $Nyears$=2 is the number of years in the simulation horizon, η=0.34 is the turbine efficiency, g = 9.81 m/s^2 is the gravitational acceleration, γ = 1000 kg/m^3 is the water density,

$\bar{h}_t^{GIII}$ is the net hydraulic head, and q_t^{turb} is the turbinated flow.

2. Final Gibe III level (i^{GIII_L}), evaluated at the end of the filling transient H_{2y} in meters above sea level [masl]:

$$i^{GIII\_L} = h^{GIII}_{\mathbb{H}_{\not\curvearrowright}} \tag{6}$$

3. Final Turkana level drop (i^{T_Ld}), evaluated in terms of absolute level drop referred to the initial lake level h_0^T:

$$i^{T\_Ld} = h^{T}_{\mathbb{H}_{\not\curvearrowright}} - h_0^T \tag{7}$$

4. Flood Pulse (i^{FP}), defined as the average annual flood pulse magnitude computed as the maximum inflow reaching the delta during the flood season of August-September:

$$i^{FP} = \frac{1}{Nyears} \left[\sum_{y=1}^{Nyears} \max_{\tau \in [Aug,Sept]} q^{delta}_{\tau,y} \right] \tag{8}$$

where he flow in the Omo delta is given by $q^{delta}_{t+1} = r^{GIII}_{t+1-lag} + q^{lateral}_{t+1}$.

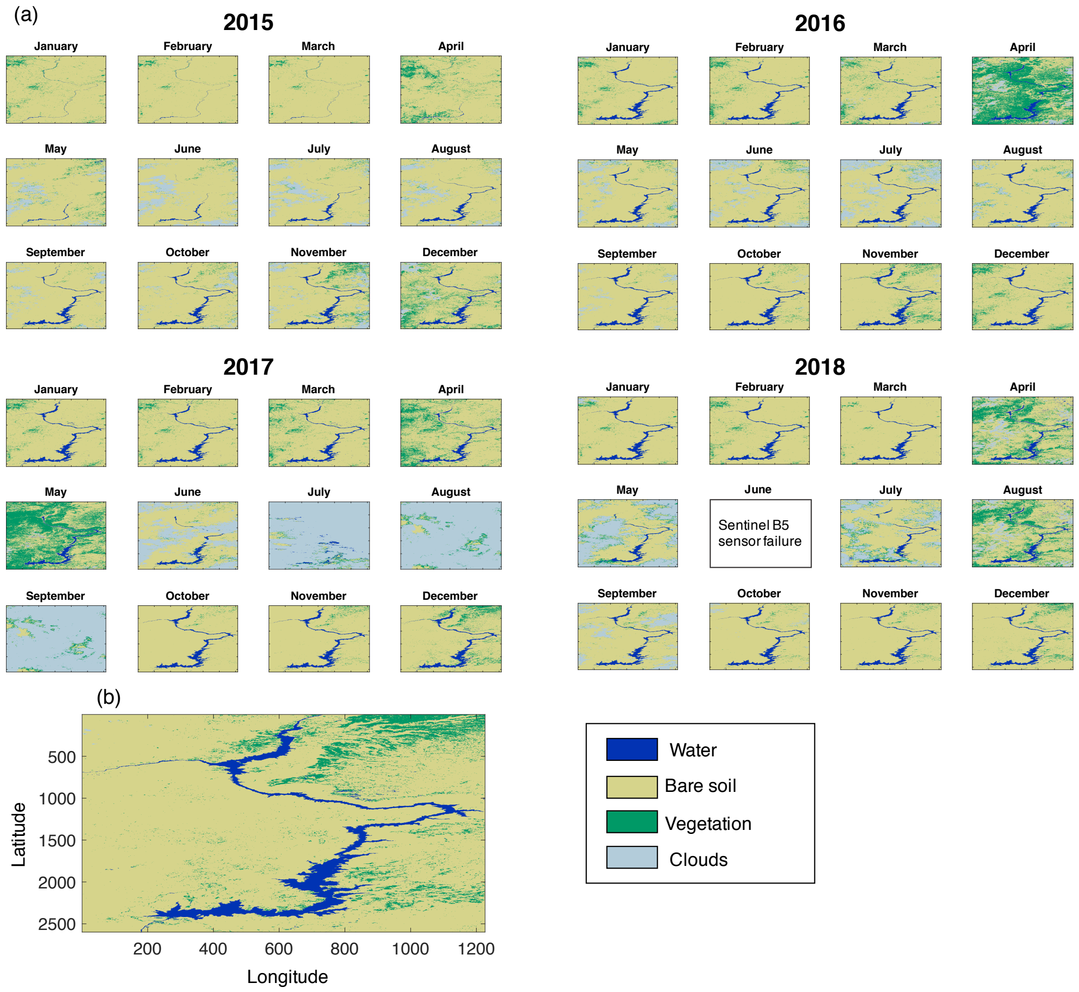

Supplementary Figure 2 Classification of water, vegetation, bare soil, and clouds at Gibe III reservoir location from Sentinel 2 satellite imagery. For the months of June to September 2017, and May to July 2018, the cloud cover was too persistent to allow composing a cloud free image; the values of adjacent months were thus interpolated. Additionally, a sensor failure temporarily interrupted Sentinel data collection via B5 sensor in the month of June 2018.

Signal	Crossvalidation Accuracy		Significance Score (%)		Location	Period
	negative	positive	negative	positive		
AMM	0.64	0.81	88.3	84.2	Atlantic	5-10 years
AMO	0.62	0.67	99.0	71.5	Atlantic	Multi-decadal
NTA	0.58	0.57	97.4	67.0	Atlantic	2-4 years
CAR	0.57	0.56	96.2	82.4	Atlantic	5-10 years
NAO	0.71	0.47	95.7	71.0	Atlantic	2-4 years
BEST	0.17	0.46	91.7	73.9	Pacific	2-4 years
MEI	0.59	0.47	63.8	91.3	Pacific	2-4 years
WP	0.39	0.61	33.3	99.9	Pacific	1-3 years
PMM	0.52	0.41	91.0	90.3	Pacific	Decadal
PNA	0.58	0.53	98.8	60.7	Pacific	2-5years
PDO	0.55	0.27	88.4	88.8	Pacific	Decadal
EPNP	0.31	0.63	66.8	94.2	Pacific	3-6 years
NP	0.44	0.52	93.1	93.6	Pacific	3-6 years
WIO	0.62	0.52	99.9	81.3	Indian	2-5years
SEIO	0.80	0.51	98.7	55.2	Indian	Decadal
DMI	0.61	0.55	98.5	97.0	Indian	2-5 years

Supplementary Table 1: Accuracy of the phase specific, univariate linear forecast models and associated significance score for the 16 tested climate signals. Model accuracy in crossvalidation is measured via the Pearson correlation coefficient. The Significance Score corresponds to the percentage of Montecarlo random shuffling trials that identify a smaller number of significantly correlated SST grid points with respect to unshuffled data.

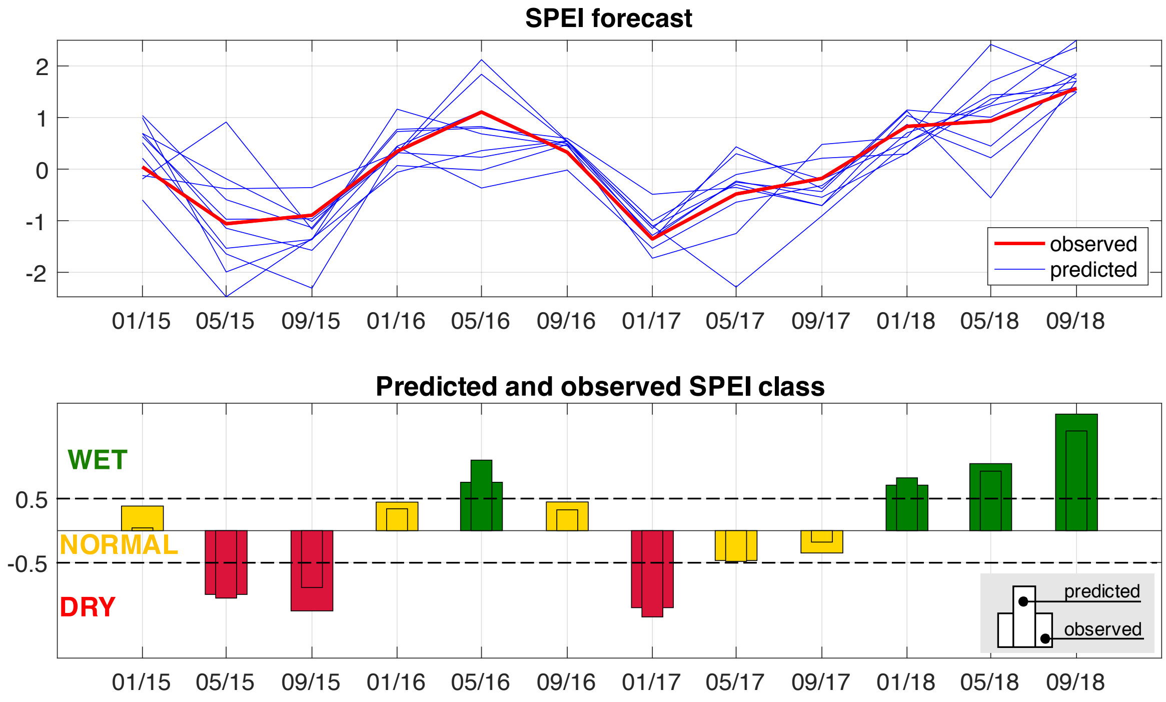

Supplementary Figure 3 SPEI forecast. Top panel reports the ensemble of teleconnection-based seasonal forecast for the Standardized Precipitation and Evaporation Index. The ensemble average is then used to classify the SPEI forecast in dry, normal, and wet conditions; this classification is correct for all the seasons.

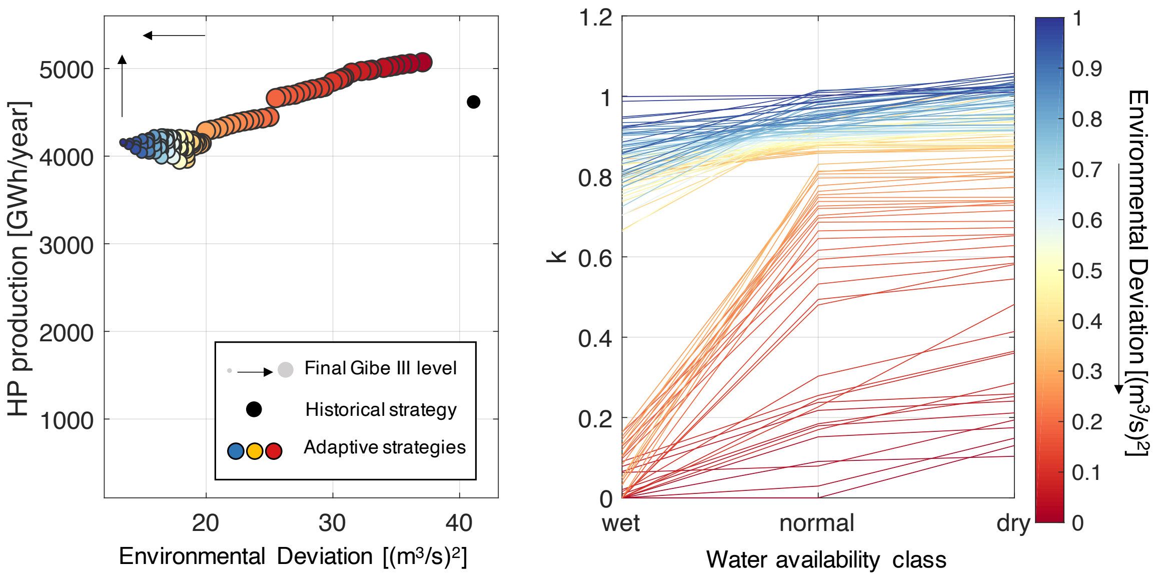

Supplementary Figure 4 Optimal adaptive filling policies. Left panel reports policies performance in the objective space, in terms of Hydropower Production (vertical axis, to be maximized), Environmental Deviation (horizontal axis, to be minimized), and Final Gibe III level (circle size, to be maximized). The historical policy (black circle) attains a comparable performance with respect to the designed policies in terms of hydropower production and Gibe III final level, but is associated to the highest environmental deviation. The climate-informed adaptive strategies demonstrate the potential to contain downstream alterations without impacting filling efficiency in terms of electricity generation or dam filling rapidity. The right panel reports the optimal values of the scaling factors associated to the three SPEI classes, which are lower for wet seasons than for dry seasons, indicating that the adaptive filling policies will release a larger water volumes when a dryer than average season is expected to avoid magnifying drought impacts on downstream activities, and

will impound a larger fraction of inflows in case of a wet spell as more abundant basin wide precipitations can support downstream activities.

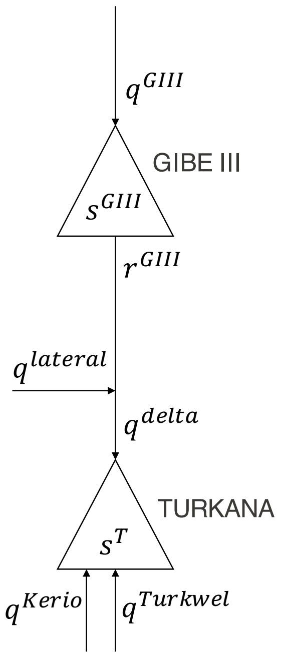

Supplementary Figure 5 Topological Scheme of the OTB: comprising Gibe III reservoir, lake Turkana, the Omo river stretch connecting the two water bodies, and the external inflows.

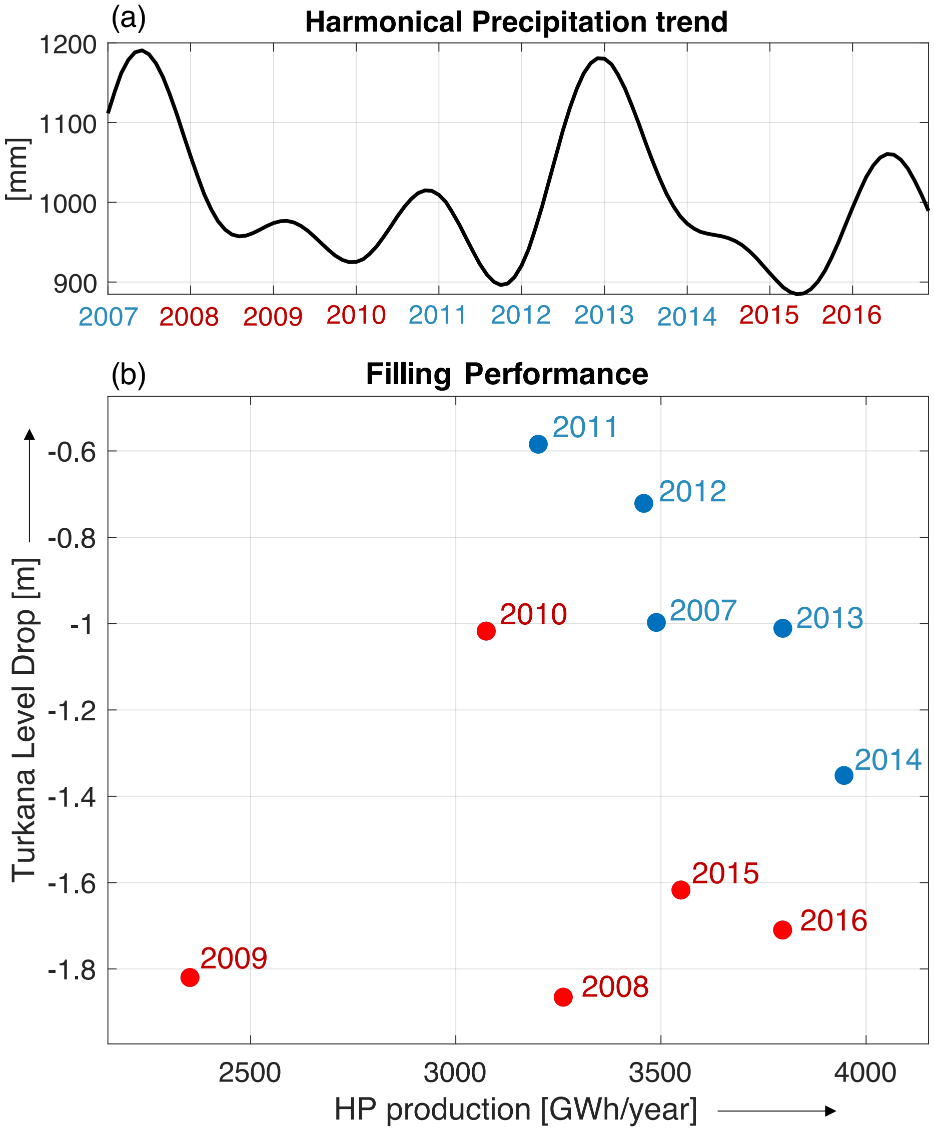

Supplementary Figure 6 : The current harmonic phase in precipitation is a strong determinant of the filling performance. We tested 10 alternative filling timing corresponding to consecutive years from 2007 to 2016, the selected time window contains two prolonged negative harmonic phases, namely 2008-2010 and 2015-2016 (panel a). The performance of alternative filling timings is shown in panel (b) in terms of the simulated

level drop in lake Turkana (vertical axis), representing downstream interests, and the HP production (horizontal axis), representing upstream interests, the arrow indicates the direction of desired performance. Results show that negative harmonic phases, colored in red, are associated with a poor filling performance, conversely, positive harmonic phases, in blue, are associated with good filling outcomes with respect to both upstream and downstream interests.

Competing Interests The authors declare that they have no competing financial interests.

References

1. Soncini-Sessa, R., Castelletti, A. & Weber, E. *Integrated and participatory water resources management: Theory* (Elsevier, Amsterdam, NL, 2007).
